# Concordant ETV6::RUNX1-positive acute lymphoblastic leukemia in monozygotic twins: a case report and review of the literature

**DOI:** 10.3389/fmed.2026.1825773

**Published:** 2026-06-18

**Authors:** Xiaohua Li, Cuicui Wang, Jianchang Li, Chaoyue Liu, Weimei Sun, Yanjun Zhang, Jian Shi

**Affiliations:** 1 Pediatric Hematology and Endocrinology, Binzhou Medical University Hospital, Binzhou, Shandong, China; 2 Department of Hepatobiliary and Pancreatic Surgery, Binzhou Medical University Hospital, Binzhou, Shandong, China; 3 Medical Department, Binzhou Medical University Hospital, Binzhou, Shandong, China

**Keywords:** two-hit, acute lymphoblastic leukemia, ETV6::RUNX1, identical twins, in utero origin

## Abstract

Patient A, a 1-year-and-6-month-old male, was admitted due to “fever for 2 days”. Patient B, a 1-year-and-9-month-old male, the monozygotic twin brother of Patient A, was admitted due to “fever for 1 day and abnormal blood findings detected 1 h prior to admission”. The twin siblings underwent bone marrow examinations and were consecutively diagnosed with acute lymphoblastic leukemia, both testing positive for the ETV6::RUNX1 fusion gene. They received chemotherapy according to the CCLG-ALL-2018 protocol for 2 years, and both achieved complete remission. Since the completion of treatment, they have remained in continuous complete remission for over 2 years of follow-up. This case report provides additional clinical evidence supporting “the in utero origin” and “two-hit” hypotheses in pediatric ALL.

## 1 Introduction

Acute lymphoblastic leukemia (ALL) is the most common malignancy in childhood, accounting for over 20% of all pediatric cancers. Concordant leukemia in monozygotic twins is a rare phenomenon, with approximately 70 reported cases in the literature worldwide. The concordance rate for leukemia in twins approaches 100% in infancy, is approximately 10% between 1 and 6 years of age, and is rarely observed at older ages (1). We report a pair of monozygotic twin toddlers, both diagnosed with B-cell acute lymphoblastic leukemia (B-ALL) and both positive for the ETV6::RUNX1 fusion gene. A review of the relevant literature was conducted to explore the mechanisms underlying leukemia development and progression. Reporting new cases of concordant leukemia in twins is encouraged to enrich the database for this rare disease.

## 2 Case report

### 2.1 Patient A

#### 2.1.1 Case presentation

A 1-year-and-6-month-old male, was the first-born twin. He was admitted to our hospital due to “fever for 2 days,” with a peak temperature of 40 °C. He was born at 34 weeks and 5 days of gestation as part of a G3P3 pregnancy. Physical examination revealed lethargy, moderate pallor, conjunctival pallor, and pale lips. Cardiopulmonary examination was unremarkable. Hepatomegaly was noted, with the liver palpable 2 cm below the right costal margin and 2.5 cm below the xiphoid process, exhibiting a firm consistency. The spleen was not palpable. Complete blood count showed: white blood cells 2.5 × 10^9^/L, red blood cells 2.7 × 10^12^/L, hemoglobin 70 g/L, platelets 144 × 10^9^/L, lymphocytes 93.6%, neutrophils 2.7%. MCV 77 fl, MCH 26 pg, MCHC 338 g/L. C-reactive protein was 81.73 mg/L. Upon admission, coagulation function, anemia workup, liver function, myocardial enzymes, biochemistry, lipid profile analysis and EBV testing were all within normal limits. Abdominal ultrasound revealed hepatosplenomegaly. Chest CT showed increased bronchovascular markings and decreased density of the cardiac chambers, suggestive of anemia, along with splenomegaly. Electrocardiogram and echocardiogram were normal. A bone marrow examination was performed. Bone marrow smear revealed morphology suggestive of acute lymphoblastic leukemia, with a markedly increased proportion of lymphocytes, of which lymphoblasts accounted for 90.5%. Immunophenotyping was consistent with acute lymphoblastic leukemia (Common B-ALL). The ETV6::RUNX1 fusion gene was positive. Karyotype analysis of bone marrow cells showed 46, XY.

#### 2.1.2 Diagnosis

Acute lymphoblastic leukemia (Common-B-ALL, low risk).

#### 2.1.3 Treatment and outcome

Chemotherapy was administered according to the CCLG-ALL-2018 protocol. At the end of induction remission, bone marrow minimal residual disease (MRD) assessment indicated complete remission. Following early intensification, quantitative detection of the ETV6::RUNX1 fusion gene was negative. Subsequent regular chemotherapy was continued per protocol. The patient experienced complications including infections, myelosuppression, gastrointestinal symptoms, and liver function abnormalities during treatment, which were managed promptly with symptomatic treatment and resolved. The total treatment duration was 2 years. Since the cessation of treatment, the patient has been followed up for nearly 2 years and 11 months and has remained in continuous complete remission based on bone marrow examinations.

### 2.2 Patient B

#### 2.2.1 Case presentation

A 1-year-and-9-month-old male, was the second-born twin and the monozygotic twin brother of Patient A. He was admitted to our hospital due to “fever for 1 day and abnormal blood findings detected 1 h prior to admission.” Physical examination revealed a generally well appearance but with an anemic facies and pale lips. Cardiopulmonary examination was unremarkable. The abdomen was soft, and neither hepatomegaly nor splenomegaly was palpable. Complete blood count showed: white blood cells 7.3 × 10^9^/L, red blood cells 3.4 × 10^12^/L, hemoglobin 93 g/L, platelets 103 × 10^9^/L, lymphocytes 97.9%, neutrophils 1%. MCV 82 fl, MCH 28 pg, MCHC 337 g/L. C-reactive protein was 40.7 mg/L. Upon admission, coagulation function, iron studies (anemia panel), liver function, myocardial enzymes, biochemistry, and lipid profile were within normal limits. EBV and TORCH serologies were negative. Abdominal ultrasound revealed mild hepatosplenomegaly. Chest CT showed minor inflammation in the upper lobe of the right lung. Head CT was unremarkable. Full abdominal CT revealed gas accumulation in some intestinal loops. Electrocardiogram and echocardiogram were normal. A bone marrow examination was performed. Bone marrow smear revealed morphology suggestive of acute lymphoblastic leukemia, with a markedly increased proportion of lymphocytes, of which lymphoblasts accounted for 96%. Immunophenotyping was consistent with acute lymphoblastic leukemia (Pre-B-ALL). The ETV6::RUNX1 fusion gene was positive. Karyotype analysis of bone marrow cells showed 46, XY.

#### 2.2.2 Diagnosis

Acute lymphoblastic leukemia (Pre-B-ALL, low risk).

#### 2.2.3 Treatment and outcome

Chemotherapy was administered according to the CCLG-ALL-2018 protocol. At the end of induction remission, bone marrow minimal residual disease (MRD) assessment indicated complete remission. Following early intensification, quantitative detection of the ETV6::RUNX1 fusion gene was negative. Subsequent regular chemotherapy was continued per protocol. The patient experienced complications including infections, myelosuppression, gastrointestinal symptoms, and liver function abnormalities during treatment, which were managed promptly with symptomatic treatment and resolved. The total treatment duration was 2 years. Since the cessation of treatment, the patient has been followed up for 2 years and 8 months and has remained in continuous complete remission based on bone marrow examinations.

The simplified clinical profiles of Patient A and Patient B are shown in Table 1.

**Table 1:** The simplified clinical profiles of Patient A and Patient B.

Patient's Name	Patient A	Patient B
Gender	Male	Male
Symptoms	Fever for 2 days	Fever for 1 day and abnormal blood findings detected 1 h prior to admission
Initial complete blood count	White blood cells 2.5 × 10^9^/L, red blood cells 2.7 × 10^12^/L, hemoglobin 70 g/L, platelets 144 × 10^9^/L, lymphocytes 93.6%, neutrophils 2.7%	White blood cells 7.3 × 10^9^/L, red blood cells 3.4 × 10^12^/L, hemoglobin 93 g/L, platelets 103 × 10^9^/L, lymphocytes 97.9%, neutrophils 1%
Immunophenotyping	Acute lymphoblastic leukemia (Common B-ALL)	Acute lymphoblastic leukemia (Pre-B-ALL)
Fusion gene	ETV6::RUNX1	ETV6::RUNX1
Diagnose	Acute lymphoblastic leukemia (Common-B-ALL, low risk)	Acute lymphoblastic leukemia (Pre-B-ALL, low risk)
Time to MRD negativity in bone marrow	The end of induction remission	The end of induction remission
Time to negativity of fusion gene	The end of early intensification	The end of early intensification
Total treatment duration	2 years	2 years
Follow-up duration	2 years and 11 months	2 years and 8 months

## 3 Discussion

Acute lymphoblastic leukemia (ALL) is classified into B-ALL and T-ALL based on immunophenotyping, with B-ALL accounting for up to 85% of cases. The ETV6::RUNX1 fusion gene, also known as TEL/AML1, is the most common translocation in childhood B-ALL, resulting from the t (12; 21) (p13; q22) chromosomal translocation. Studies have reported that the prevalence of the ETV6::RUNX1 fusion gene among children with ALL in China ranges from 20 to 25% (2, 3). The ETV6::RUNX1 fusion gene has been identified as a favorable prognostic marker in patients with leukemia (4). Numerous clinical studies have confirmed that pediatric ALL patients harboring the ETV6::RUNX1 fusion gene exhibit distinct clinical and biological characteristics: they typically present with low to normal white blood cell counts, demonstrate high sensitivity to chemotherapeutic agents (particularly L-asparaginase), and have an excellent long-term disease-free survival rate (5). In the present cases, both twin siblings presented with white blood cell counts within the normal range or slightly decreased at initial diagnosis. They received treatment according to the Chinese “Diagnosis and Treatment Guidelines for Childhood Acute Lymphoblastic Leukemia” (2018 edition), achieving early complete remission and MRD negativity. The chemotherapy course lasted approximately 2 years, and during follow-up since treatment discontinuation, both patients have remained in continuous complete remission with negative bone marrow MRD and quantitative gene testing for nearly 3 years. The favorable treatment response and long-term disease-free survival observed in these two patients typify the excellent prognosis associated with ETV6::RUNX1-positive ALL. These findings are consistent with current research reports on the prognosis of ETV6::RUNX1-positive ALL.

In the present cases, both twin siblings developed the disease during early childhood, were diagnosed with ALL, with an interval of 3 months between diagnoses, and shared ETV6::RUNX1 positivity. This finding suggests a common clonal origin for the leukemia in these monozygotic twins. According to reports, the incidence of concordant acute leukemia in monozygotic twins ranges from 5 to 25%, while it is extremely rare in dizygotic twins (6). The phenomenon of concordant childhood leukemia occurring in twins was first documented in the German medical literature in 1882 (7). By the mid-20th century, several case reports of concordant leukemia in twins had been published. Review articles published in the 1960s and 1970s proposed the concept of the pre-natal origin of childhood leukemia (7, 8). Most scholars propose that the in utero monoclonal origin hypothesis and the mechanism of intraplacental transfer are the primary explanations for concordant leukemia in monozygotic twins (9). The proposed mechanism involves a random genetic translocation occurring in a hematopoietic precursor cell in one fetus, generating a “pre-leukemic” clone. Due to the shared single placenta (monochorionic) in monozygotic twins, placental vascular anastomoses provide a pathway for cellular exchange. This founder cell carrying the pre-leukemic clone disseminates through the placental circulation and successfully engrafts into the hematopoietic system of the other fetus (7, 10, 11).

This hypothesis has been supported by several landmark studies. For instance, Ford et al., through retrospective analysis of neonatal dried blood spots from twins, confirmed the presence of identical ETV6::RUNX1 sequences at birth, providing direct evidence for an in utero origin (12). The identification of identical fusion events in monozygotic twins both affected by leukemia supports the concept of a common clonal origin, as the probability of two twins independently acquiring leukemic events with identical breakpoints is exceedingly low. Ford and colleagues first demonstrated this in three pairs of monozygotic twins with infant acute lymphoblastic leukemia harboring KMT2A rearrangements, where each twin pair exhibited KMT2A restriction fragments of identical size (13). Subsequently, several pairs of monozygotic twins with acute lymphoblastic leukemia and identical gene fusion events have been identified, including ETV6::RUNX1 (12, 14, 15), BCR-ABL1 (16) and TCF3-ZNF384 translocations (17). In the present cases, the time interval between disease onset in the two siblings was three months. By the time Patient B developed the disease, Patient A had already achieved molecular negativity, rendering the initial bone marrow samples unavailable for analysis. Additionally, neither patient had cord blood samples preserved at birth. Consequently, it was not possible to confirm through sequencing that the fusion gene breakpoints in the leukemic cells of the two siblings were identical, which would have provided further strong evidence supporting the in utero origin hypothesis. The absence of breakpoint sequencing prevented definitive confirmation of a shared leukemic clone. Due to the low incidence of leukemia in monozygotic twins and the limited number of cases, conducting sequencing studies is challenging because of factors such as the lack of preserved neonatal cord blood samples and the asynchronous onset of disease in twin pairs. There is an urgent need for more cases and more in-depth gene sequencing to provide clinical data and support for research. In the future, establishing a collaborative registry for twin leukemias and promoting the preservation of neonatal genetic samples could provide further theoretical support for understanding the mechanisms of leukemogenesis.

Many childhood leukemias, particularly ALL with specific fusion genes such as ETV6::RUNX1, have their initiating events traceable to the fetal developmental period in utero (18). In neonatal studies, the prevalence of children harboring a ETV6::RUNX1 pre-leukemic clone is approximately 1%, yet only about 1% of such children ultimately develop leukemia (19). The presence of ETV6::RUNX1-positive clonal cells has also been detected in healthy adults, with an incidence of approximately 0.5%. These observations collectively indicate that the fusion gene alone is insufficient to cause leukemia, leading to the proposition of the “two-hit” hypothesis of leukemogenesis (19): a latent period exists between the pre-leukemic state in fetal life and the onset of overt leukemia. An initial genetic mutation (such as the formation of the ETV6::RUNX1 fusion gene) occurs during early embryonic development, resulting in both twins carrying this congenital abnormality in their hematopoietic cells. After birth, under the influence of environmental factors (such as viral infections or physical/chemical stimuli), one twin may acquire a second genetic mutation (e.g., RAS gene mutation, P53 inactivation) that triggers leukemia. The other twin, sharing an identical genetic background, is pre-disposed to a similar second hit under comparable environmental exposures, leading to disease manifestation. In the present cases, both twins concurrently carried the ETV6::RUNX1 fusion gene, suggesting that this abnormality likely originated during the embryonic period. Combined with the clinical data—the relatively short interval between disease onsets and the similar clinical presentations—these findings further support the synergistic role of shared genetic susceptibility and similar environmental exposures.

This case highlights important considerations regarding surveillance strategies in monozygotic twins. When one monozygotic twin is diagnosed with ALL (especially ETV6::RUNX1-positive), surveillance of the healthy co-twin should not be limited to simple notification. We recommend adopting a proactive surveillance strategy: Immediate comprehensive physical examination and complete blood count (CBC) testing; Health education for parents regarding warning symptoms of leukemia, such as fatigue, bleeding, bone pain, and fever; Under professional medical guidance, although standardized surveillance protocols are lacking, periodic clinical evaluation and CBC monitoring may be considered in selected high-risk situations. Although bone marrow aspiration screening in healthy children remains controversial regarding ethics and benefits, maintaining a high index of suspicion is absolutely essential (20). In the present case, Patient B presented with fever; given his sibling's history of ALL, when his CBC showed lymphocytosis and anemia, infection could not be considered as the primary cause without further investigation. Active diagnostic workup to exclude leukemia was necessary to achieve early diagnosis, timely treatment, and avoid missed diagnosis.

This article presents a typical case of monozygotic twins concordant for ETV6::RUNX1-positive acute lymphoblastic leukemia, further supporting the “in utero origin hypothesis” and the “two-hit hypothesis” of leukemogenesis from both clinical and molecular perspectives, thereby reaffirming the status of TEL-AML1 as a favorable prognostic marker. This case reminds clinicians that when confronted with twin leukemia patients, they should be equipped with the scientific acumen to discern universal principles from specific phenomena, while simultaneously providing individualized and health management plans for the healthy co-twin. Ultimately, this approach ensures that the value of rare cases can benefit a broader population of pediatric patients.

## Data Availability

The original contributions presented in the study are included in the article/supplementary material, further inquiries can be directed to the corresponding author.

## References

[1] Khera S Kurup A Agarwal S Tripathi P. Synchronous presentation of *ETV6::RUNX1* fusion positive concordant B-acute lymphoblastic leukaemia in identical twin toddlers. BMJ Case Rep. (2023) 16:e257139. doi: 10.1136/bcr-2023-257139 37967932 PMC10660162

[2] Jawdat D Almashaqbeh W Sumaily A Albaloushi N Jammah S Alsultan A. Screening for pre-leukemia TEL-AML1 chromosomal translocation in banked cord blood units: cord blood bank perspective. Cell Tissue Bank. (2020) 21:625–30. doi: 10.1007/s10561-020-09855-y 32812094

[3] Jedraszek K Malczewska M Parysek-Wójcik K Lejman M. Resistance mechanisms in pediatric B-cell acute lymphoblastic leukemia. Int J Mol Sci. (2022) 23:3067. doi: 10.3390/ijms23063067 35328487 PMC8950780

[4] Organista-Nava J Gómez-Gómez Y Illades-Aguiar B Leyva-Vázquez MA. Regulation of the miRNA expression by TEL/AML1, BCR/ABL, MLL/AF4 and TCF3/PBX1 oncoproteins in acute lymphoblastic leukemia (review). Oncol Rep. (2016) 36:1226–32. doi: 10.3892/or.2016.4948 27431573

[5] Hematology Group Pediatrics Branch Chinese Medical Association Editorial Editorial Board of the Chinese Journal of Pediatrics. Guidelines for the diagnosis and treatment of childhood acute lymphoblastic leukemia (2018 edition). Chin J Pediatr. (2019) 57:9–21. Available online at: https://www.pcscn.org.cn/

[6] de Smith AJ Spector LG. In utero origins of acute leukemia in children. Biomedicines. (2024) 12:236. doi: 10.3390/biomedicines12010236 38275407 PMC10813074

[7] Greaves MF Maia AT Wiemels JL Ford AM. Leukemia in twins: lessons in natural history. Blood. (2003) 102:2321–33. doi: 10.1182/blood-2002-12-3817 12791663

[8] Macmahon B Levy MA. Prenatal origin of childhood leukemia evidence from twins. N Engl J Med. (1964) 270:1082–5. doi: 10.1056/NEJM196405212702102 14121487

[9] Kotecha RS Murch A Kees U Cole CH. Pre-natal, clonal origin of t (1; 11) (p32; q23) acute lymphoblastic leukemia in monozygotic twins. Leuk Res. (2012) 36:46–50. doi: 10.1016/j.leukres.2011.03.014 21474181

[10] Lewi L Deprest J Hecher K. The vascular anastomoses in monochorionic twin pregnancies and their clinical consequences. Am J Obstet Gynecol. (2013) 208:19–30. doi: 10.1016/j.ajog.2012.09.025 23103301

[11] Teuffel O Betts DR Dettling M Schaub R Schäfer BW Niggli FK. Prenatal origin of separate evolution of leukemia in identical twins. Leukemia. (2004) 18:1624–9. doi: 10.1038/sj.leu.2403462 15356660

[12] Ford AM Bennett CA Price CM Bruin MC Van Wering ER Greaves M. Fetal origins of the TEL-AML1 fusion gene in identical twins with leukemia. Proc Natl Acad Sci U S A. (1998) 95:4584–8. doi: 10.1073/pnas.95.8.4584 9539781 PMC22533

[13] Ford AM Ridge SA Cabrera ME Mahmoud H Steel CM Chan LC . In utero rearrangements in the trithorax-related oncogene in infant leukaemias. Nature. (1993) 363:358–60. doi: 10.1038/363358a0 8497319

[14] Wiemels JL Ford AM Van Wering ER Postma A Greaves M. Protracted and variable latency of acute lymphoblastic leukemia after TEL-AML1 gene fusion in utero. Blood. (1999) 94:1057–62. doi: 10.1182/blood.V94.3.1057.415k10_1057_1062 10419898

[15] Hong D Gupta R Ancliff P Atzberger A Brown J Soneji S . Initiating and cancer-propagating cells in TEL-AML1-associated childhood leukemia. Science. (2008) 319:336–9. doi: 10.1126/science.1150648 18202291

[16] Cazzaniga G van Delft FW Lo Nigro L Ford AM Score J Iacobucci I . Developmental origins and impact of BCR-ABL1 fusion and IKZF1 deletions in monozygotic twins with Ph+ acute lymphoblastic leukemia. Blood. (2011) 118:5559–64. doi: 10.1182/blood-2011-07-366542 21960589 PMC3217357

[17] Bueno C Ballerini P Varela I Menendez P Bashford-Rogers R. Shared D-J rearrangements reveal cell of origin of TCF3-ZNF384 and PTPN11 mutations in monozygotic twins with concordant BCP-ALL. Blood. (2020) 136:1108–11. doi: 10.1182/blood.2020006604 32609826

[18] Greaves M. A causal mechanism for childhood acute lymphoblastic leukaemia. Nat Rev Cancer. (2018) 18:471–84. doi: 10.1038/s41568-018-0015-6 29784935 PMC6986894

[19] Mori H Colman SM Xiao Z Ford AM Healy LE Donaldson C . Chromosome translocations and covert leukemic clones are generated during normal fetal development. Proc Natl Acad Sci U S A. (2002) 99:8242–7. doi: 10.1073/pnas.112218799 12048236 PMC123052

[20] Schüz J Erdmann F. Environmental exposure and risk of childhood leukemia: an overview. Arch Med Res. (2016) 47:607–14. doi: 10.1016/j.arcmed.2016.11.017 28476188

